# Generalized trust and all-cause mortality: A population-based prospective cohort study

**DOI:** 10.1177/14034948251340480

**Published:** 2025-09-22

**Authors:** Martin Lindström, Mirnabi Pirouzifard

**Affiliations:** Social medicine and Health Policy, Department of Clinical Sciences and Centre for Primary Health Care Research, Lund University, Malmö, Sweden

**Keywords:** Generalized trust in other people, social trust, social participation, social support, social capital, all-cause mortality, Sweden

## Abstract

**Aims:**

Social science literature suggests that unlimited trust in others may lead to adverse outcomes, and that moderate trust may be more optimal in social interactions. The aim was to analyze associations between generalized trust in other people and all-cause mortality using a four-alternative generalized trust item that includes moderate trust.

**Study design:**

Prospective cohort study.

**Methods:**

The 2008 Public Health Survey in Scania, southern Sweden, was conducted with a postal questionnaire followed by three reminders, with 28,198 respondents **(**54.1% response rate**)**. The survey was linked to all-cause mortality register data with 9.3-year follow-up. Multiple Cox regression analyses were performed.

**Results:**

The item “Most people can be trusted” yielded 5.5% “Completely agree,” 57.6% “Agree,” 29.5% “Don’t agree” and 7.4% “Don’t agree at all.” Covariates of age, sex, socioeconomic status, chronic disease, mental health, health-related behaviors, social participation and social support showed significant bivariate associations with all-cause mortality. All-cause mortality remained significantly lower in the moderately high trust category (“Agree”) compared to the very high trust reference category (“Completely agree”) in the multiple analyses. In contrast, all-cause mortality in the low and very low trust categories did not significantly differ from the very high trust reference. Trust was also dichotomized into high trust (“Completely agree”/“Agree”) versus low trust. Dichotomized low trust showed significantly higher all-cause mortality only when sex and age were included as covariates in the model.

**Conclusions:**

**The construction, use and interpretation of generalized trust items should be scrutinized, and moderate generalized trust in others investigated as a health protective factor.**

## Introduction

Associations between social capital and mortality have been investigated since the mid-1990s [1,2]. A major theoretical approach to social capital in public health defines social capital as social participation**/**social networks, reciprocity, generalized trust in other people and trust in the institutions of society that enhance collaboration between actors and facilitate transactions [3,4,5]. Results of empirical studies indicate that social participation in social networks displays the strongest and most consistent statistical associations with mortality. Generalized trust in other people is also associated with lower mortality [6]. However, the construct of items and the number of answer alternatives utilized in questionnaires to assess generalized trust remain an important issue because, for theoretical reasons, moderate generalized trust may be more health protective than both unlimited trust and low trust/lack of trust. Generalized trust in others has mostly been analyzed dichotomously as high versus low trust [6], but the association between trust and health may not always be linear.

Social science literature suggests that unlimited trust in others may lead to adverse social, financial and other outcomes, and that moderate trust may be more optimal in social interactions [7]. Unconditional and unlimited generalized trust in other people may often prove problematic. The consequences for individuals, groups or entire societies may be serious or even catastrophic if erroneous decisions are made without open debates and discussions that entail both mutual trust and sound levels of criticism and questioning. Democracy rests on sound questioning and evaluation of arguments, not unlimited and uncritical trust in opponents [7]. Unless cooperation based on trust also serves a component of egoistic motivation, the practice of cooperation runs the risk of yielding unstable and untenable results [8]. Consequently, a social order that is not grounded in at least some self-interest will tend to be unpredictable and unstable, which is the reason why particularly unlimited trust is not always functional [9]. The public health and healthcare system literature also suggests that unconditional trust in the healthcare system and cognitive laziness may lead to heuristic behavior and the utilization of cognitive shortcuts, which may reduce the cost of retrieving necessary access and treatment information that eventually lead**s** to poorer health outcomes [10]. Recent studies show that the groups with moderate vertical trust across a power gradient in the healthcare system [11] and in politicians responsible for the healthcare system [12] displayed significantly lower all-cause mortality throughout multiple survival analyses than the very high institutional trust category in the region of Scania in southernmost Sweden.

The notion tested in the present study is that moderate horizontal generalized trust in other people without a power gradient may also be optimal in relation to all-cause mortality. Psychological and psychosocial stress, norms regarding health-related behaviors, access to healthcare and amenities and exposure to crime may all be plausible mechanisms [1]. One example of these plausible mechanisms concerns economic fraud and crime. In recent decades, crime in the form of digital fraud and fraud by telephone directed against the elderly has surged in Sweden. The reluctance to report exposure to crime may not only stem from fear of retaliation from the perpetrators or reluctance to be in contact with the police authorities but also from the perception of shame among victims [13]. The “dark side of social capital” has been discussed in terms of the adverse effects of social exclusion on health [14], but an additional aspect of this phenomenon may be the shame experienced by fraud victims with previously very high trust that hinders reporting to the police. Financial exploitation victimization of the elderly is associated with mortality, hospitalization and poor somatic and mental health [15]. Sweden has traditionally been a high trust society, and older generations have for several decades displayed higher levels of generalized trust [16].

This study investigates associations between generalized trust in others and all-cause mortality using the item “Most people can be trusted” with four alternative answers, which presents the possibility to separate moderate trust from very high (unlimited) trust. For comparison, we also analyze in separate model**s** the commonly used dichotomy high versus low trust and all-cause mortality. Relevant covariates age, sex, socioeconomic status (SES), country of birth, chronic disease, mental health, leisure-time physical activity (LTPA), smoking, alcohol consumption, social participation and social support are adjusted for in the Cox regression models.

The aim is to investigate associations between generalized trust in other people and all-cause mortality in survival analyses, including a 9.3-year follow-up, adjusting for relevant covariates. The generalized trust item will be analyzed both with all four scale alternatives with very high trust as reference but also dichotomized as high versus low trust.

## Material and methods

### Study population

In 2008, a public health questionnaire was sent to a stratified random sample of the official register population in the age interval 18–80 years living in Scania, the southernmost part of Sweden. The questionnaire was sent with a postal invitation letter. It was also possible to answer the questionnaire online. Three reminders were sent to initial non-responders. 28,198 people in the original sample responded, which was a 54.1% response rate.

*Region Skåne*, the authority responsible for healthcare in southernmost Sweden, financed and administered the survey. The questionnaire contains 134 items including demographics, social and work conditions, social capital, social support, health-related behaviors and self-rated health. The stratification of the random sample was based on 30 city parts in four major cities and 29 smaller municipalities. Statistics Sweden (*Statistikmyndigheten*) generated the stratified sample and created the population weight, which accounts for the stratification by compensating for lower response rates in some demographic groups. The baseline questionnaire data from 2008 was linked to prospective mortality data obtained from the National Board of Health and Welfare (*Socialstyrelsen*). The linking was possible by using the Swedish 10-digit personal identification numbers, and these were provided to *Socialstyrelsen* in cooperation with a third party (private company). After linkage conducted by *Socialstyrelsen*, the survival data was sent to the research group without possibilities of individual identification.

Ethical consent was granted from the Ethical Committee (*Etikprövningsnämnden*) in Lund (No. 2010/343).

### Outcome

Mortality was followed prospectively from August 27–November 14, 2008, (depending on registration date of respondents’ answers) until December 31, 2017 (9.3 years onward), or until death. A total of 24,659 participants were included in this study, 11,142 men and 13,517 women, which excludes 3,403 respondents with internally missing values on one or more items. In addition, loss to follow-up was recorded for 136 participants. All-cause mortality was analyzed.

### Independent variables

*Generalized trust in other people* was assessed with the statement “Most people can be trusted,” which included the alternative answers “Don’t agree at all” (very low trust), “Don’t agree” (low trust), “Agree” (high trust) and “Completely agree” (very high trust). The item was analyzed in two ways. First, it was analyzed with all four alternatives with “Completely agree” as reference. Second, it was dichotomized with the two first alternatives together as “low trust” and the two latter as “high trust,” with high trust as reference.

*Sex*: Men and women were collapsed in all analyses.

*Age* was treated as a continuous variable.

*Country of birth* included born in Sweden and born abroad.

*SES* was defined as non-manual employees in high, medium and low positions, skilled and unskilled manual workers, and self-employed/farmers. The categories outside the workforce include the unemployed (job seekers), students, early retired (younger than 65), long-term sick leave, pensioners (65 and above) and unclassified.

*Chronic disease* was obtained with the question “Do you have any long-term disease, ailment or injury, any disability or other weakness?” Alternatives were “Yes” and “No.”

*Mental health* was assessed with the GHQ12 instrument, which entails 12 items on psychological health including anxiety, depression, sleep disturbance, loss of confidence, loss of ability to perform daily activities and loss of ability to cope with everyday problems during the past five weeks. If three or more of the 12 subitems indicated poor psychological health, mental health was defined as poor.

*LTPA* was assessed with the alternatives regular exercise (at least three times per week at least 30 minutes/occasion, causing sweating), moderate regular exercise (exercising once or twice per week at least 30 minutes/occasion, causing sweating), moderate exercise (walking, cycling or equivalent activity status in leisure-time less than two hours walking, cycling or equivalent activity/week) and sedentary status (less than two hours walking or equivalent activity/week). The three active alternatives were defined as high LTPA and the sedentary alternative as low LTPA.

*Smoking* was assessed with the question “Do you smoke?” which included alternatives daily, non-daily and non-smoker. The two latter alternatives were collapsed, which yielded “daily smoking” and “not daily smoking/no smoking.”

*Alcohol consumption* includes the question “How often have you consumed alcohol during the past twelve months?” including alternatives “4 times per week or more,” “2–3 times per week,” “2–4 times per month,” “Once per month or more seldom” and “Never.”

*Social participation* includes visits at least once during the past year to a study circle/course at a workplace, other study circle/course, union meeting, other organization meeting, theatre/cinema, arts exhibition, church, sports event, letter to editor of newspaper/journal, demonstration, night club/entertainment, gathering of relatives and private party. A fourteenth alternative was none of the above. Three or fewer activities were defined as low social participation.

*Social support* was measured by the item “Do you know one or some persons who could give you proper personal support to cope with the stress and problems of life?” with alternatives “Yes, absolutely certain,” “Yes, probably,” “Not certain” and “No.” The first alternative was defined as high social support and the other three as low.

All independent variables and their associations with generalized trust in other people are presented in Table I.

**Table I.** Descriptive characteristics (%) of age, sex, socioeconomic status (SES), country of birth, chronic disease, mental health, leisure-time physical activity, daily smoking, alcohol consumption, social participation and social support according to generalized trust in other people. The 2008 Public Health Survey in Scania, Sweden. Total population ***n* = 24,659**. Weighted prevalence.

	**(Generalized trust in other people)**				
	Completely agree	Agree	Don’t agree	Don’t agree at all	*p*-value
	*n* = 1451	*n* = 14,696	*n* = 6869	*n* = 1643	
	5.5%	57.6%	29.5%	7.4%	
**Age**, yrs: mean ±SD^a^	47.4±16.4	46.8 ± 16.2	44.1± 17.5	44.1 ± 18.5	<0.001
**Sex^b^**					
Men	51.6	50.6	47.6	51.6	<0.001
Women	48.4	49.4	52.4	48.4	
**Socioeconomic status (SES)^b^**					<0.001
High non-manual	15.9	11.3	4.5	3.3	
Medium non-manual	18.4	16.6	10.1	5.7	
Low non-manual	5.2	8.6	8.0	6.0	
Skilled manual	7.1	10.1	12.1	10.5	
Unskilled manual	8.6	11.1	15.3	17.1	
Self-employed/farmer	6.3	6.2	6.0	4.4	
Early retired	2.9	2.8	5.0	6.8	
Unemployed	2.8	2.7	5.4	8.9	
Student	8.1	7.0	10.6	9.9	
Old-age pensioner	20.1	18.1	15.1	16.1	
Unclassified	3.8	4.7	6.5	8.0	
Long-term sick leave	0.8	0.8	1.4	3.3	
**Born outside Sweden^b^**	17.5	14.8	20.6	28.1	<0.001
**Chronic disease^b^**	24.1	25.6	32.3	38.7	<0.001
**Poor mental health^b^**	12.3	13.7	23.4	31.6	
**Low leisure-time physical activity^b^**	12.2	11.5	16.3	22.1	<0.001
**Daily smoking^b^**	10.1	11.8	18.0	23.5	<0.001
**Alcohol drinking during the past year^b^**					<0.001
Never	13.2	8.8	14.0	21.1	
Once a month or more seldom	17.2	21.4	25.2	30.0	
2–4 times a month	31.7	37.6	35.4	28.4	
2–3 times a week	29.0	24.8	18.9	14.5	
At least 4 times a week	8.9	7.5	6.5	6.0	
**Low social participation^b^**	33.2	34.6	44.6	60.2	<0.001
**Low social support^b^**	20.1	29.6	40.7	48.0	<0.001

a *p*-value: Independent samples ANOVA test, 2-tailed.

b *p*-value: Pearson Chi-Square test, 2-sided.

### Statistics

Prevalence (%) of all items were displayed based on the four alternatives of the generalized trust in other people item. Differences between the four alternatives were analyzed using a chi-square test for categorical variables (all variables except age) and an ANOVA test for continuous items (age) (*p*-values).

Hazard rate ratios (HRRs) with 95% confidence intervals (95% CIs) of all-cause mortality were calculated in bivariate survival (Cox regression) analyses for both the four alternatives and dichotomized trust in other people variables and all other independent variables (Table II).

**Table II.** Hazard rate ratios (HRRs) with 95% confidence intervals (95% CIs) of all-cause mortality in bivariate analyses. The 2008 Public Health Survey in Scania with 9.3 years follow-up (2008–2017). Men and women combined. Total population ***n* = 24,659**. Weighted.

	Reference	Crude		Number of deaths
		HRR	(95% CI)	
**All causes of death**				1470
**Generalized trust in other people**				
High trust (“Agree”)	Very high trust	**0.74★**	(0.58–0.93)	
Low trust (“Don’t agree”)		0.81	(0.63–1.03)	
Very low trust (“Don’t agree at all”)		1.00	(0.72–1.38)	
**Generalized trust in other people** (dichotomized)				
Low trust	High trust	1.12	(0.98–1.26)	
**Sex**	Men	**0.56★★★**	(0.49–0.64)	
**Age**		**1.11★★★**	(1.10–1.12)	
**Socioeconomic status**	High non-manual			
Medium non-manual		0.98	(0.57–1.69)	
Low non-manual		1.09	(0. 57–2.11)	
Skilled manual		1.71	(0.95–3.07)	
Unskilled manual		1.10	(0.60–2.00)	
Self-employed/farmer		1.18	(0.61–2.30)	
Early retired		**11.09★★★**	(6.57–18.71)	
Unemployed		1.65	(0.82–3.30)	
Student		**0.21★★**	(0.06–0.69)	
Old-age pensioner		**17.40★★**	(10.82–27.98)	
Unclassified		0.59	(0.20–1.76)	
Long-term sick leave		**8.67★★★**	(4.54–16.56)	
**Country of birth**	Sweden	0.83	(0.68–1.01)	
**Chronic disease**	No	**2.98★★★**	(2.63–3.39)	
**Poor mental health**	Good	**1.25★★**	(1.07–1.47)	
**Low leisure-time physical activity**	High	**2.50★★★**	(2.18–2.86)	
**Daily smoking**	No	**1.51★★★**	(1.28–1.78)	
**Alcohol drinking past year**	Never			
Once a month or more seldom		**0.74★★**	(0.61–0.89)	
2–4 times a month		**0.36★★★**	(0.29–0.44)	
2–3 times a week		**0.56★★★**	(0.46–0.68)	
At least 4 times a week		**1.29★**	(1.03–1.61)	
**Low social participation**	High	**3.25★★★**	(2.84–3.71)	
**Low social support**	High	**1.28★★★**	(1.12–1.46)	

Significance levels: ★ *p*<0.05, ★★ *p*<0.01, ★★★ *p*<0.001. Weighted hazard ratios. Bootstrap method (1000 replicates) for variation estimation.

HRRs with 95% confidence intervals (95% CIs) of all-cause mortality by generalized trust were analyzed in survival (Cox regression) analyses with all four alternatives of the generalized trust item with the “Completely agree” alternative as reference. Seven models were analyzed: model 0 unadjusted, model 1 adjusted for age and sex, model 2 additionally adjusted for SES, country of birth, chronic disease and poor mental health, and model 3 additionally adjusted for LTPA, tobacco smoking and alcohol consumption. In model 4 social participation was added, in model 5 social support was added and in model 6 both social participation and social support were added to the other variables in model 3 (Table III).

**Table III.** Hazard rate ratios (HRRs) with 95% confidence intervals (95% CIs) of all-cause mortality according to generalized trust in other people (four alternatives). The 2008 Public Health Survey in Scania with 9.3 years follow-up (2008–2017). Men and women combined. Total population ***n* = 24,659**. Weighted.

	Model 0		Model 1		Model 2		Model 3		Model 4		Model 5		Model 6		Number of deaths
	HRR	(95% CI)	HRR	(95% CI)	HRR	(95% CI)	HRR	(95% CI)	HRR	(95% CI)	HRR	(95% CI)	HRR	(95% CI)	
**All causes of death**															1470
Very high trust	1.00		1.00		1.00		1.00		1.00		1.00		1.00		
High trust	**0.74★**	(0.58–0.93)	0.79	(0.62–1.01)	**0.76★**	(0.60–0.95)	**0.78★**	(0.63–0.97)	**0.78★**	(0.62–0.97)	**0.79★**	(0.63–0.98)	**0.79★**	(0.63–0.99)	
Low trust	0.81	(0.63–1.03)	1.06	(0.82–1.36)	0.87	(0.68–1.11)	0.87	(0.68–1.10)	0.86	(0.68–1.09)	0.88	(0.69–1.12)	0.87	(0.69–1.11)	
Very low trust	1.00	(0.72–1.38)	1.23	(0.90–1.68)	0.94	(0.69–1.28)	0.88	(0.65–1.20)	0.86	(0.63–1.17)	0.89	(0.65–1.21)	0.87	(0.64–1.19)	

Model 0 unadjusted.

Model 1 adjusted for sex and age.

Model 2 additionally adjusted for socioeconomic status, country of birth, chronic disease and mental health.

Model 3 additionally adjusted for leisure-time physical activity, daily smoking and alcohol consumption.

Model 4 adjusted for sex, age, socioeconomic status (SES), country of birth, chronic disease, mental health, leisure-time physical activity, daily smoking, alcohol consumption and social participation.

Model 5 adjusted for sex, age, socioeconomic status (SES), country of birth, chronic disease, mental health, leisure-time physical activity, daily smoking, alcohol consumption and social support.

Model 6 adjusted for sex, age, socioeconomic status (SES), country of birth, chronic disease, mental health, leisure-time physical activity, daily smoking, alcohol consumption, social participation and social support.

Significance levels: ★ *p*<0.05, ★★ *p*<0.01, ★★★ *p*<0.001. Weighted hazard ratios. Bootstrap method (1000 replicates) for variation estimation.

HRRs with 95% confidence intervals (95% CIs) of all-cause mortality by generalized trust in other people were also analyzed in survival (Cox regression) analyses using dichotomized trust with high trust as reference. The same seven models (0–6 as previously described) were calculated (see Table IV).

**Table IV.** Hazard rate ratios (HRRs) with 95% confidence intervals (95% CIs) of all-cause mortality according to dichotomized generalized trust in other people (high/low). The 2008 Public Health Survey in Scania with 9.3 years follow-up (2008–2017). Men and women combined. Total population ***n* = 24,659**. Weighted.

	Model 0		Model 1		Model 2		Model 3		Model 4		Model 5		Model 6		Number of deaths
	HRR	(95% CI)	HRR	(95% CI)	HRR	(95% CI)	HRR	(95% CI)	HRR	(95% CI)	HRR	(95% CI)	HRR	(95% CI)	
**All causes of death**															1470
High trust	1.00		1.00		1.00		1.00		1.00		1.00		1.00		
Low trust	1.12	(0.98–1.26)	**1.35★★★**	(1.19–1.53)	1.14	(1.00–1.30)	1.09	(0.95–1.24)	1.07	(0.94–1.22)	1.09	(0.96–1.25)	1.08	(0.95–1.23)	

Model 0 unadjusted.

Model 1 adjusted for sex and age.

Model 2 additionally adjusted for socioeconomic status, country of birth, chronic disease and mental health.

Model 3 additionally adjusted for leisure-time physical activity, daily smoking and alcohol consumption.

Model 4 adjusted for sex, age, socioeconomic status (SES), country of birth, chronic disease, mental health, leisure-time physical activity, daily smoking, alcohol consumption and social participation.

Model 5 adjusted for sex, age, socioeconomic status (SES), country of birth, chronic disease, mental health, leisure-time physical activity, daily smoking, alcohol consumption and social support.

Model 6 adjusted for sex, age, socioeconomic status (SES), country of birth, chronic disease, mental health, leisure-time physical activity, daily smoking, alcohol consumption, social participation and social support.

Significance levels: ★ *p*<0.05, ★★ *p*<0.01, ★★★ *p*<0.001. Weighted hazard ratios. Bootstrap method (1000 replicates) for variation estimation.

**Figure 1.**
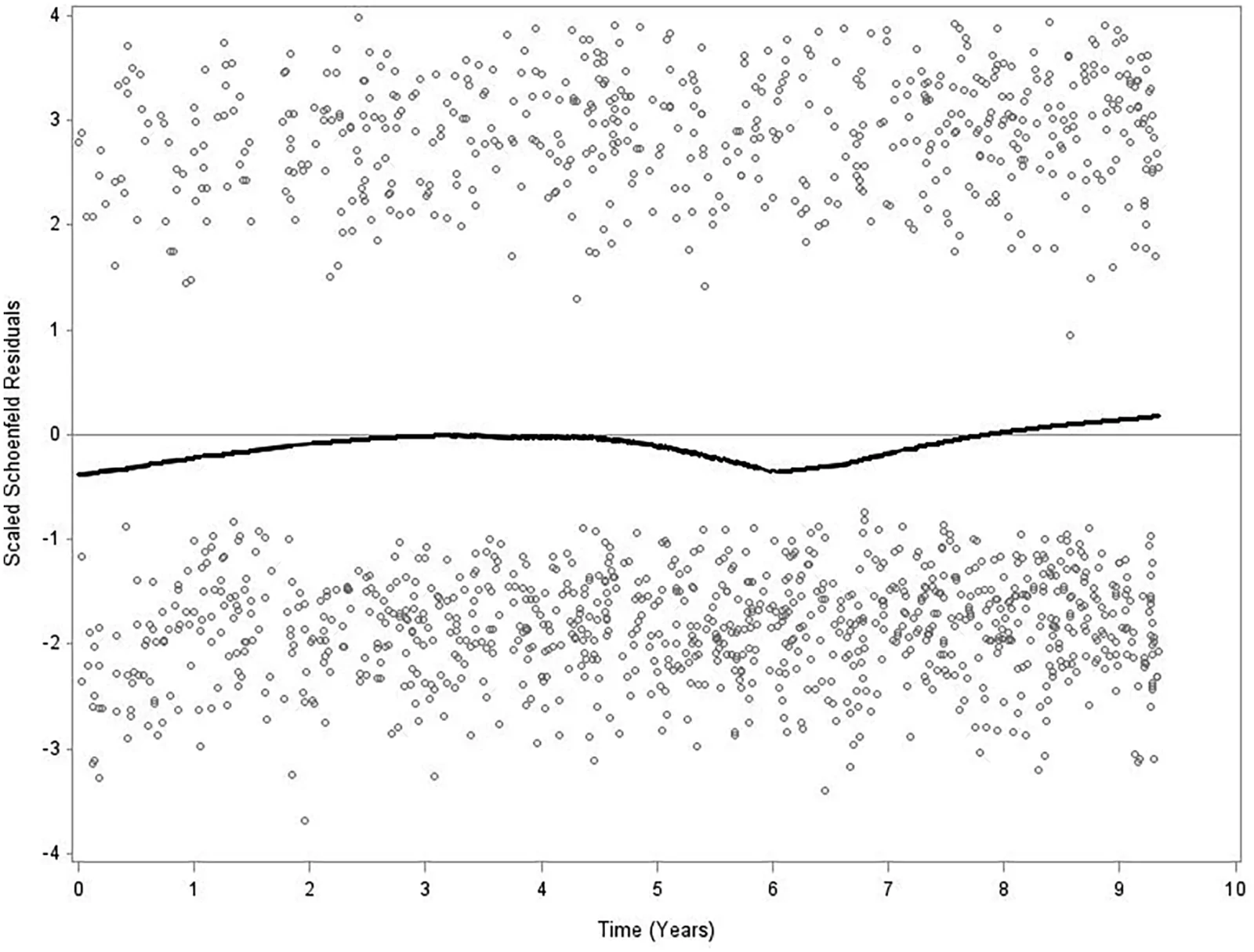
Schoenfeld residuals of generalized trust in other people (dichotomized into low and high groups) and all-cause mortality. The interaction term between dichotomized generalized trust in other people and time with regard to all-cause mortality across the 9.3-year period showed a statistically not significant *p* = 0.886, which indicates proportionality. Men and women, *n* = 24,659. The Public Health Survey in Scania 2008 with 9.3 years follow-up (2008–2017).

Follow-up time in days from baseline to death or last follow-up date (2017-12-31) were assessed. Analysis of sampling variability without distributional assumptions is possible by using bootstrap analysis [17]. Bootstrap analysis with 1000 iterations to obtain confidence intervals and *p*-values was conducted to assure accurate variance estimation on weighted data. Proportionality testing was conducted for the association over time between generalized trust and all-cause mortality. The proportional hazards assumption was calculated by the introduction of an interaction term between time and generalized trust. Schoenfeld residuals were calculated for generalized trust (dichotomized generalized trust) and all-cause mortality (Figure 1). The SAS software version 9.4 was used in all analyses.

## Results

A 5.5% proportion had very high trust, 57.6% high trust, 29.5% low trust and 7.4% very low trust. Respondents with low and very low trust were younger than those with very high and high trust (*p*<0.001). Men had somewhat higher trust than women (*p*<0.001). Respondents with higher SES had higher trust than respondents with lower SES. Pensioners had higher trust and students lower (*p*<0.001). Respondents born in other countries than Sweden had lower trust than the respondents born in Sweden (*p*<0.001). Respondents with chronic disease and respondents with poor mental health displayed lower trust (*p*<0.001). Respondents with low LTPA and respondents who were daily smokers displayed lower trust than their reference categories (both *p*<0.001). Respondents with less frequent alcohol consumption reported lower trust than respondents with more frequent alcohol consumption (*p*<0.001). Both low social participation and low social support were significantly associated with low trust (*p*<0.001).

The bivariate survival analyses show that moderately high trust had significantly lower HRR 0.74 (0.58–0.93) of all-cause mortality than very high trust. The low trust and very low trust categories did not significantly differ from the very high trust alternative. The dichotomized low and high generalized trust categories did not significantly differ in all-cause mortality. Women had significantly lower all-cause mortality. The risk of all-cause mortality increased with age. The early retired, the unemployed, old-age pensioners and the group on long-term sick leave had significantly higher HRRs of all-cause mortality than the non-manual employees in higher positions reference, while students had a significantly lower HRR 0.21 (0.06–0.69). The categories born in Sweden and abroad did not significantly differ. Chronic disease, poor mental health, low LTPA and daily smoking displayed significantly higher HRRs of all-cause mortality than their respective references. The categories with alcohol consumption once a month or more seldom, 2–4 times/month and 2–3 times/week had significantly lower all-cause mortality than the never alcohol consumption reference. Low social participation HRR 3.25 (2.84–3.71) and low social support 1.28 (1.12–1.46) were also significantly associated with all-cause mortality.

The moderately high trust category had significantly lower HRRs of all-cause mortality than the very high trust category in model 0 and models 2–6. In model 2 adjusted for age, sex, SES, country of birth, chronic diseases and mental health, the HRR was 0.78 (0.63–0.97), in model 3 additionally adjusted for LTPA, daily smoking and alcohol consumption the HRR was 0.78 (0.63–0.97) and in model 6 additionally adjusted for both social participation and social support the HRR was 0.79 (0.63–0.99). For the low trust and very low trust categories, all-cause mortality did not significantly differ from the very high trust reference category in models 0–6.

The dichotomized low trust category had a significantly higher HRR 1.35 (1.19–1.53) of all-cause mortality than the dichotomized high trust reference category in model 1, adjusted for age and sex. In model 2, the HRR 1.14 (1.00–1.30) of all-cause mortality for dichotomized low trust was no longer statistically significant, and it remained not significant in models 3–6.

The Schoenfeld residuals for generalized trust and all-cause mortality were stable over time when dichotomized high trust was compared with dichotomized low trust. The interaction term for generalized trust in other people and all-cause mortality was not statistically significant, *p* = 0.886, which indicates proportionality.

## Discussion

The results show that moderately high trust displays significantly lower all-cause mortality over a 9.3-year follow-up period than the very high trust reference in the multiple adjusted models. In contrast, no statistically significant differences in all-cause mortality were observed for the low and very low trust categories compared to the very high trust reference. The results also display significantly higher all-cause mortality for the dichotomized low trust category compared to the dichotomized high trust category in the model adjusted for age and sex, but not in the further multiple adjusted models. It seems that collapsing the high and very high trust categories into high dichotomized trust weakens the difference in all-cause mortality between high and low dichotomized trust. The results lead us to methodologically question the use of dichotomization as well as the number of fixed answer alternatives in the measurement of generalized trust.

The finding that moderate high generalized trust in others may be a possible health protective factor even compared to very high trust under certain social circumstances increases the importance of discussion of trust measurements and their number of fixed answer alternatives. Trust measurements with a variety of formulated questions and statements have existed for decades [18]. The construction and number of answer alternatives, which range from two [19] to at least five [20], should be discussed. The construction, use and interpretation of measurements for the assessment of generalized trust should follow valid criteria [21] when moderate high trust is investigated as a protective factor for health.

The main point is that measurements of generalized trust in other people that only include two or few (e.g. “yes,” “no” and “don’t know”) alternatives miss the opportunity to assess associations with and plausible effects of moderate high trust on health outcomes. The main issue here is not that measurements may be phrased in various ways or that several measurements may be combined into indexes [22]. Instead, the main issue is that measurements have different numbers of alternatives. A common number of alternatives is three with the options “Yes” (trust), “It depends” and “No” [23,24,25], or “High,” “Moderate” and “Low” levels of trust/social capital [16]. Other items include four alternatives (“Most people can be trusted”) with e.g. alternatives “Completely agree,” “Agree,” Don’t agree” and “Don’t agree at all,” although mostly analyzed dichotomized into high and low trust [26], and others five alternatives [18]. Some international surveys include scales measuring generalized trust; for example, the European Social Survey (ESS) includes a scale 0–10 for the measurement of trust [27]. Some studies only have alternatives “Yes” as a depiction of high social trust, with just one more alternative indicating “No” [19]. This study indicates that trust measurements should include sufficient numbers of alternatives to assess the health effects of moderate trust in others. Such items would optimally include at least four alternative answers. This should be elaborated in future studies as well as social circumstances that promote moderate trust as a health protective factor.

Social participation, which is a central aspect of social capital, and social support, which is included in one strand of social capital theory [6], were introduced in the analyses but did not affect the significant association between moderate trust and all-cause mortality.

### Strengths and limitations

This longitudinal population-based study has sufficient statistical power to indicate 20–30% lower all-cause mortality in the moderate trust (“Agree”) category compared to the very high (“Completely agree”) reference category. The 54.1% response rate is tolerable given response rates of other surveys in the same period. The survey population shows good representativeness when it comes to sex, age, birth country and education.

For decades, the literature has presented several examples of items designed to capture generalized trust [18]. SES was measured by occupation and relationship to the labor market. SES was not measured regarding education and income, because the education item in the survey has a considerably higher number of internally missing values and the survey does not contain income. GHQ12 has been defined and validated elsewhere [28]. The LTPA item has been validated [29]. Survey items regarding smoking are valid. The validity of the social participation and social support items have been discussed elsewhere [6]. All-cause mortality register data has a very high validity in Sweden [30].

The results of this study are most likely generalizable primarily to Scania although the trust level has been consistently high in Sweden just as in Scania since the late 1990s [31], but prolonged queueing for healthcare and exposure to criminality may vary in Sweden. The results are probably not completely generalizable to Denmark, Finland and Norway for the additional reason that Sweden has for more than a decade exhibited somewhat lower generalized trust and lower trust in politicians compared to the other Nordic countries, according to the European Social Survey [27].

Adjustment for relevant covariates and confounders was conducted in the multiple analyses in Tables III and IV. Personality trait items were not included in the survey.

## Conclusion

Moderate generalized trust in others displayed significantly lower all-cause mortality than very high generalized trust throughout the multiple survival analyses. The construction, use and interpretation of items for the assessment of generalized trust should be more thoroughly investigated.
